# Integrated periodontal pathogens and circulating miRNAs: a novel non-invasive panel of biomarkers for pancreatic cancer

**DOI:** 10.3389/fcimb.2025.1678114

**Published:** 2025-10-31

**Authors:** Zeinab Hesami, Valerio Pazienza, Meysam Olfatifar, Amir Sadeghi, Samira Mohammadi-Yeganeh, Nadia Trivieri, Hesameddin Eghlimi, Mojdeh Hakemi-Vala, Elena Binda, Hamidreza Houri

**Affiliations:** ^1^ Department of Microbiology, School of Medicine, Shahid Beheshti University of Medical Sciences, Tehran, Iran; ^2^ Foodborne and Waterborne Diseases Research Center, Research Institute for Gastroenterology and Liver Diseases, Shahid Beheshti University of Medical Sciences, Tehran, Iran; ^3^ Gastroenterology Unit IRCSS Casa Sollievo della Sofferenza, Opera di San Pio da Pietrelcina, San Giovanni Rotondo/FG, Italy; ^4^ Gastroenterology and Hepatology Diseases Research Center, Qom University of Medical Sciences, Qom, Iran; ^5^ Gastroenterology and Liver Diseases Research Center, Research Institute for Gastroenterology and Liver Diseases, Shahid Beheshti University of Medical Sciences, Tehran, Iran; ^6^ Department of Medical Biotechnology, School of Advanced Technologies in Medicine, Shahid Beheshti University of Medical Sciences, Tehran, Iran; ^7^ Cancer Stem Cells Unit, Institute for Stem Cell Biology, Regenerative Medicine and Innovative Therapeutics (ISBReMIT), IRCSS Casa Sollievo della Sofferenza, Opera di San Pio da Pietrelcina, San Giovanni Rotondo/FG, Italy; ^8^ Department of General Surgery, Ayatollah Taleghani Hospital, Shahid Beheshti University of Medical Science, Tehran, Iran; ^9^ Celiac Disease and Gluten Related Disorders Research Center, Research Institute for Gastroenterology and Liver Diseases, Shahid Beheshti University of Medical Sciences, Tehran, Iran

**Keywords:** pancreatic cancer, periodontal pathogens, circulating miRNAs, non-invasive biomarkers, cancer diagnosis

## Abstract

**Introduction:**

The associations between oral bacterial pathogens and the risk of pancreatic cancer (PC) have been reported in several epidemiological studies. In this study, we evaluated the diagnostic potential of periodontal pathogens *Porphyromonas gingivalis* and *Aggregatibacter actinomycetemcomitans* in combination with circulating oncomiRNAs, including miR-21 and miR-155.

**Methods:**

A total of 41 PC patients and 40 age- and sex-matched controls were recruited for the study. The salivary bacterial load of *P. gingivalis* and *A. actinomycetemcomitans*, along with the copy number of miR-21 and miR-155 in blood, were measured using quantitative real-time PCR. Subsequently, logistic regression and receiver operating characteristic (ROC) analysis were used to determine the association of biomarkers with PC risk and their diagnostic performance, respectively.

**Results:**

Elevated load of the periodontal pathogens *P. gingivalis* in females (OR = 2.31; 95% CI 0.98-5.47) and *A. actinomycetemcomitans* in diabetic individuals (OR = 3.66; 95% CI 0.47-6.68) was associated with a higher risk of PC. Moreover, the diagnostic model incorporating two salivary species and two circulating miRNAs demonstrated an area under the curve (AUC) of 0.878 (95% CI 0.802-0.955).

**Discussion:**

This study offers compelling new evidence supporting the idea that the combined analysis of salivary microbiota and circulating miRNAs serves as an informative avenue for the discovery of non-invasive biomarkers for PC, potentially applicable to early detection and clinical screening.

## Introduction

Pancreatic cancer (PC) is an extremely lethal disease, characterized by high mortality within five years of diagnosis. GLOBOCAN estimates that by 2040, the global incidence and mortality rates of PC could increase by over 75% and 80%, respectively (Ferlay et al., 2021; Hesami et al., 2024). The unfavorable prognosis of PC is largely due to its tendency to quickly spread to the lymphatic system and distant organs (Li et al., 2004; Whitcomb, 2004; Hezel et al., 2006). The aggressive biological behavior of PC, coupled with its high resistance to conventional chemotherapies and the absence of effective sensitive diagnostic biomarkers, results in a 5-year survival rate of only 5% for individuals diagnosed with the disease (Ries et al., 2007; Jemal et al., 2008). Approximately 15% to 20% of PC patients present with a disease that is eligible for surgical resection. However, of these patients, only about 20% achieve a 5-year survival rate (Li et al., 2004). PC stems from a complex interplay of etiological factors, with cigarette smoking being widely recognized as the only established modifiable risk factor. However, evidence also points to a potential link between diabetes, obesity, and insulin resistance with an elevated risk of developing this malignancy (Rawla et al., 2019; Wood et al., 2019). Furthermore, the strong correlation between chronic pancreatitis (CP) and a significantly heightened risk of PC suggests that inflammation may play a pivotal role in the initiation and progression of the disease. Inflammatory pathways may drive cellular proliferation and mutagenesis, hinder the ability to adapt to oxidative stress, promote angiogenesis, inhibit apoptosis, and elevate the secretion of pro-inflammatory signaling molecules (Ohara et al., 2022).

The oral cavity serves as a significant reservoir of microorganisms, encompassing over 700 species, collectively known as the oral microbiome (Aas et al., 2005). Mounting evidence indicates that oral microbiota plays crucial roles in human health, influencing immune regulation, carcinogen metabolism, and nutrient processing (Zhang et al., 2019). Periodontitis, an inflammatory condition of the oral cavity caused by dysbiosis of the oral microbiota, has been linked to an elevated risk of PC in several longitudinal studies (Hujoel et al., 2003; Stolzenberg-Solomon et al., 2003; Michaud et al., 2007). Additionally, recent epidemiological studies have shown that poor oral health and the presence of circulating and salivary antibodies to specific oral pathogens are associated with an increased risk of PC (Michaud et al., 2007; Alkharaan et al., 2020; Yu et al., 2022). Further prospective investigations have also underscored the potential of periodontal pathogens as biomarkers for the early detection of PC (Fan et al., 2018; Petrick et al., 2022). The association between periodontal pathogens and pancreatic cancer is supported by plausible biological mechanisms that extend beyond epidemiological correlation. As keystone pathogens, *Porphyromonas gingivalis* and *Aggregatibacter actinomycetemcomitans* can instigate oral dysbiosis and sustained local inflammation, which facilitates their systemic dissemination. This systemic presence can potentiate a pro-tumorigenic environment through both the induction of a persistent, low-grade inflammatory response and the direct production of carcinogenic compounds, including nitrosamines (Yu et al., 2022). Furthermore, a direct oncogenic mechanism has been proposed for *P. gingivalis* via its secretion of peptidylarginine deiminase (PPAD), an enzyme implicated in processes that may foster the acquisition of driver mutations in genes such as KRAS and TP53 (Öğrendik, 2015; Öğrendik, 2017).

MicroRNAs are short RNA molecules, typically 18–24 nucleotides in length, that have been evolutionarily conserved (Chang and Mendell, 2007). These molecules regulate the stability and translation of target mRNAs by binding to complementary sequences in the 3' untranslated regions (3' UTRs). This regulatory mechanism is crucial for maintaining cellular homeostasis and supporting developmental processes. In recent years, the significant role of miRNAs in controlling cell growth, their involvement in cancer, and their presence in cell-free biofluids (e.g., blood, plasma, serum, saliva, tears, urine, stool, pancreatic juice, breast milk, cerebrospinal fluid, and peritoneal/pleural fluids) have become increasingly apparent (Seyhan, 2023). These circulating miRNAs display specific expression patterns that correlate with changes in physiological conditions and the presence of disease. Numerous studies have identified various miRNAs with dysregulated expression linked to PC, highlighting their potential as circulating biomarkers for the diagnosis of the disease at early stages (Metcalf, 2024; Khorsand et al., 2025). In this study, we conducted a comprehensive analysis comparing the load of *P. gingivalis* and *A. actinomycetemcomitans* in salivary samples with the copy number of miR-21 and miR-155 in blood samples from both patients diagnosed with PC and control subjects, using quantitative real-time PCR. Additionally, we assessed the performance and translational potential of these periodontal pathogens and circulating miRNAs as potential biomarkers for the non-invasive detection of PC.

## Materials and methods

### Study population and sample collection

This study was reviewed and approved by the Institutional Ethical Review Committee of the School of Medicine at Shahid Beheshti University of Medical Sciences (No.IR.SBMU.MSP.REC.1402.184). In this case-control study conducted between 2022 and 2024 at Taleghani Hospital in Tehran, Iran, participants were prospectively enrolled following standardized protocols for the collection, processing, and storage of biological samples. Individuals over 18 years of age with suspected PC, confirmed through pathology diagnosis during endoscopic ultrasound (EUS) procedures, were included as cases. Controls, matched for age, sex, and hospital, were chosen from the orthopedic ward inpatients without hepatobiliary conditions or a history of cancer. Participants were excluded if they specifically had a history of chronic gastrointestinal (GI) inflammatory disorders, including inflammatory bowel diseases (IBD) or irritable bowel syndrome (IBS). Written informed consent was obtained from participating wards and study participants, respectively. Demographic data, including age, sex, body mass index (BMI), tobacco and opium use, etc., were collected during face-to-face interviews through a structured questionnaire. Clinical and paraclinical information was retrieved from hospital charts, including the stage of the disease, past medical history, past surgery history, social history, and laboratory findings. Before the sampling process, all the volunteers were instructed not to eat for 1 h prior to saliva sample collection. Unstimulated saliva samples were collected between 8:00 AM and 5:00 PM by passive drooling until a minimum volume of 500 µL was obtained. The samples were then collected, aliquoted, and preserved in RNALater and stored at 4 °C overnight, then stored at −70°C until DNA extraction. Blood samples were processed immediately on the same day of sample collection for RNA extraction and cDNA synthesis.

### Sample processing

Salivary samples were thawed, and DNA was extracted and purified using the E.Z.N.A.^®^ MicroElute^®^ Genomic DNA Kit (Omega Bio-tek, Georgia, USA). miRNA extraction from whole blood was performed using the SanPrep Column microRNA Miniprep Kit (Bio Basic, Markham, ON, Canada), followed by cDNA synthesis via RevertAid^®^ RT enzyme (Invitrogen, Thermo Fisher Scientific) and RT stem-loop primers according to established protocols (Mohammadi-Yeganeh et al., 2013). The yield and purity of the isolated DNA and RNA were assessed using a spectrophotometer (Nanodrop, Thermo Fisher Scientific, Waltham, MA, USA).

### Bacterial strains

The standard strains of *P. gingivalis* (ATCC33277) and *A. actinomycetemcomitans* (ATCC24523) were obtained from the School of Public Health, Tehran University of Medical Sciences, and Dental School, Shahid Beheshti of Medical Sciences, respectively. These strains were cultured on brucella agar supplemented with 10% sheep blood, hemin, and menadione under anaerobic conditions (5% CO2, 10% H2, 85% N2) at 37 °C. Bacterial DNA extraction was carried out from purified colonies of each strain using the DNA QIAamp DNA Mini Kit (QIAGEN, Hilden, Germany) following the provided instructions.

### Serial dilution preparation and quantitative real-time PCR

For absolute quantification of the bacterial biomarker candidates, serial dilutions were prepared to obtain standard curves based on the genomic size of standard strains and the optical density (OD) of extracted DNA. In addition, synthesized nucleotide sequences of miR-21 and miR-155 were provided to prepare serial dilutions based on fragment size and molar mass per base pair. Sequences of the oligonucleotide primers used for real-time qPCR are shown in **Supplementary Table S1**. Real-time qPCR was carried out in duplicate in reaction volumes of 10 µl using RealQ plus 2X Master Mix Probe High ROX (Ampliqon, Herlev, Denmark). The thermal cycling conditions consisted of an initial denaturation for 15 min at 95°C, followed by 40 cycles of amplification with three steps: 95°C for 20 seconds, then either 62°C for 30 seconds, and 72°C for 50 seconds for *P. gingivalis*, or 56°C for 30 seconds and 72°C for 40 seconds for *A. actinomycetemcomitans*. Additionally, miRNAs were amplified in two steps of 95°C for 15 s and 60°C for 50 s. All qPCR tests were performed using LightCycler^®^ (Roche Life Science, Berlin, Germany). The specificity of the qPCR amplification for each target was confirmed by both a single peak in the melt curve analysis and the presence of a single band of the expected size on an agarose gel. The efficiency (E) of each assay was determined from the standard curve using the formula E = 10 ^(-1/slope)^. All reactions demonstrated high efficiency, with E-values between 90% and 105% and correlation coefficients (R²) greater than 0.990, indicating sensitive and reliable quantification.

### Statistical analysis

The assumption of normality for the bacterial load and miRNA copy number data was assessed visually using quantile-quantile (Q-Q) plots. Subsequently, differences in these variables between the PC group and the non-cancer control group were evaluated through an independent samples *t*-test. Association of two microbial data, as well as the copy number of two circulating miRNAs to subject status, were assessed using the 'adonis2' implementation of PERMANOVA and distance-based redundancy analysis. To investigate the relationship between these biomarkers and PC risk, a least absolute shrinkage and selection operator (LASSO) logistic regression method was applied to estimate odds ratios (ORs) and their corresponding 95% confidence intervals (CIs). Features selected during the LASSO process were included in risk assessment analyses within stratified subgroups of cases and controls. Subsequently, receiver operating characteristic (ROC) curves were generated, and the area under the curve (AUC) was determined through numerical integration via both univariable and multivariable logistic regression models. All statistical analyses were conducted using R version 4.3.2.

## Results

### Characteristics of the study participants

As shown in **Table 1**, of the included PC patients (n=41), 68% were male, and the majority of them were more likely to be current smokers (39%) and have type 2 diabetes (61%). Among patients diagnosed with PC, most of the tumors were well differentiated (G_1_) and located in the head of the pancreas (77.1%).

**Table 1:** Distribution of demographic parameters among pancreatic cancer cases and non-cancer controls.

Variables	Characteristics	PC group	Non-cancer group	*p-value* ^*^
		n=41	n=40	
Demographic data				
Age (years)	Mean ± SD	62.68 ± 14.63	62.52 ± 14.20	0.972
Sex (%)	Female	31.7	35	0.816
	Male	68.3	65	
BMI (Kg/m^2^)	Mean ± SD	22.38 ± 4.06	24.71 ± 3.80	0.009
Cigarette smoking status (%)	Non-smoker	53.7	77.5	0.051
	Former smoker	7.3	7.5	
	Current smoker	39	15	
Smoking per day (%)	10<	9.8	17.5	0.005
	11-20	31.7	5	
	21-30	2.4	0	
	>30	2.4	0	
Addiction (%)	Not-addicted	65.9	85	0.084
	Former addicted	4.9	0	
	Current addicted	29.3	15	
Alcohol consumption (%)	No	82.9	87.5	0.040
	Former consumption	12.2	0	
	Yes	4.9	12.5	
Past medical history				
Diabetes mellitus (%)	No	20	90	<0.001
	Yes	61	39	
Newly diagnosed diabetes^┼^ (%)	No	24.4	5	<0.001
	Yes	53.7	2.5	
Cancer (%)	No	95.1	100	0.494
	Yes	4.9	0	
Hypertension (%)	No	58.5	80	0.054
	Yes	41.5	20	
Cardiovasular diseases (%)	No	75.6	90	0.140
	Yes	24.2	10	
Herniae history	No	75.6	100	0.001
	Yes	24.2	0	
Prostate diseases (%)	No	78	100	0.002
	Yes	22	0	
GI disorders (%)	No	51.2	90	<0.001
	Yes	48.8	10	
Hyperlipidemia (%)	No	85.4	77.5	0.404
	Yes	14.6	22.5	
Kidney stone (%)	No	85.4	92.5	0.482
	Yes	14.6	7.5	
Hemorrhoids (%)	No	90.2	100	0.116
	Yes	9.8	0	
Past medication history				
PPIs (%)	No	48.8	82.5	0.002
	Yes	51.2	17.5	
Insulin (%)	No	85.4	97.5	0.109
	Yes	14.6	2.5	
Metformin (%)	No	78	92.5	0.116
	Yes	22	7.5	
Levothyroxine (%)	No	78	95	0.048
	Yes	22	5	
Losartan (%)	No	78	77.5	0.379
	Yes	22	12.5	
Atorvastatin (%)	No	73.2	79.5	0.798
	Yes	26.8	22.5	
NSAIDs (%)	No	73.2	82.5	0.424
	Yes	26.8	17.5	
Tumor characteristics				
Grade (%)	G_1_	53.8		
	G_2_	42.3		
	G_3_	3.9		
Location of tumor (%)	Head	77.1		
	Neck	20		
	Body	14.3		
	Tail	5.7		
	Uncinate	5.7		
	Ampulla of vater	22.8		
	Distal	5.7		
Metastasis stage	Distal metastasis	16.7		
	Duodenal wall invasion	36.1		
	Lymphovasuclar invasion	80.5		
	Pertineural invasion	63.9		
	No metastasis	0		

^*^ *p*-values were based on t-test for continuous data, and χ^2^ test or Fisher's exact test for categorical data.

^┼^ Diagnosed in less than two years.

BMI, body mass index; PPIs, proton pump inhibitors; NSAIDs, non-steroidal anti-inflammatory drugs; GI disorders, gastrointestinal disorders.

### Salivary and blood markers differences among groups

**Figure 1:**
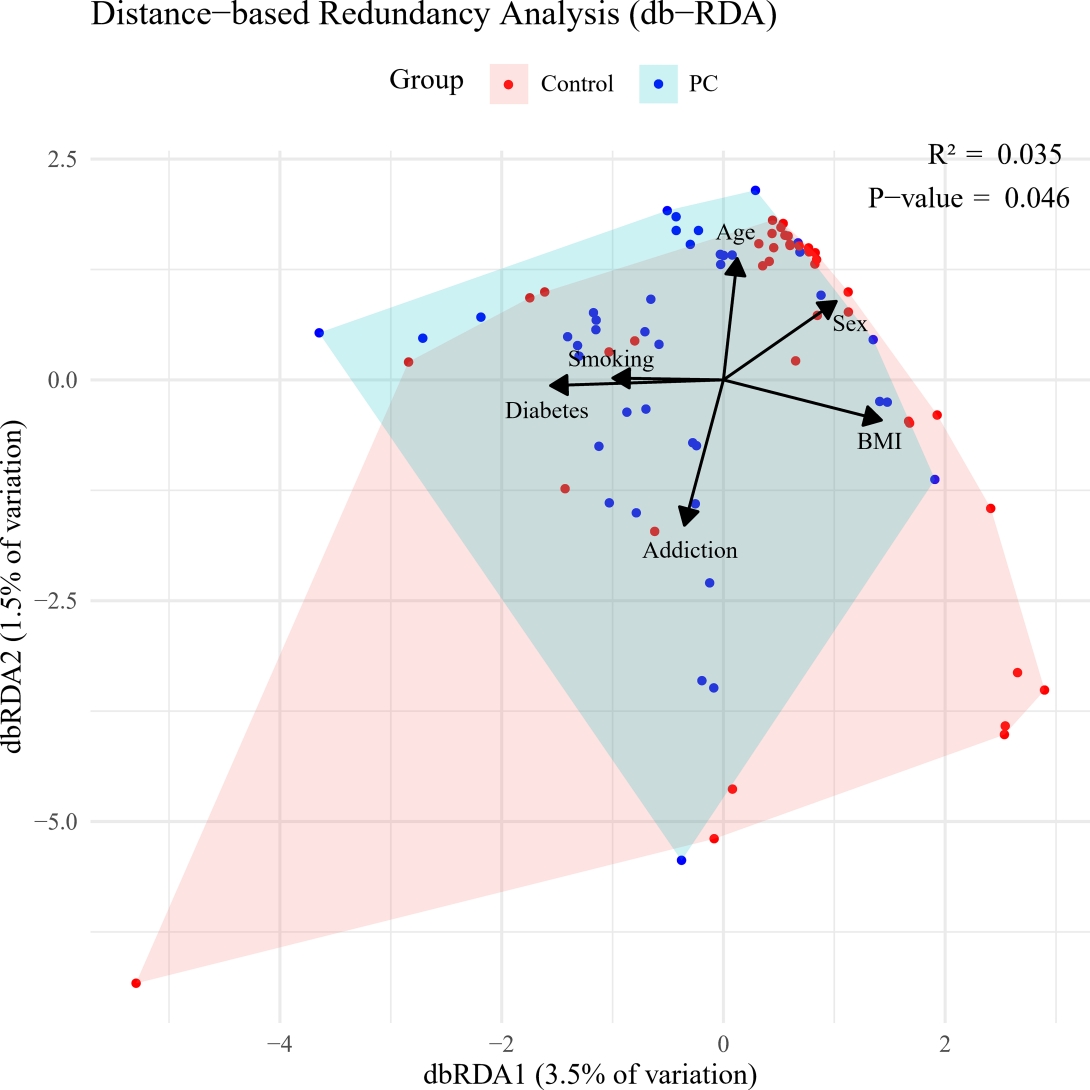
Bray-Curtis distance-based redundancy analysis (dbRDA) based on salivary loads of *Porphyromonas gingivalis* and *Aggregatibacter actinomycetemcomitans*, along with circulating miR-21 and miR-155 copy number variation, in pancreatic cancer (PC) versus control groups.

*P. gingivalis* was more prevalent in saliva from patients than controls. It was detected in approximately 61% (25 out of 41) of the patient group but was found only in 22.5% (nine out of 43) of the controls. Furthermore, a significant difference was observed in the number of *P. gingivalis* cells in salivary samples between patients and controls (*p* = 0.034). Similarly, *A. actinomycetemcomitans* was more prevalent among patients, although no significant difference was found between the two groups (*p* = 0.128). Additionally, miR-155 was overexpressed, with a significant increase (*p* = 0.034) in copy numbers observed in patients with PC compared to non-cancer individuals. Conversely, despite the overexpression of miR-21 in the PC group, no statistically significant distinctions were found between the two groups (*p* = 0.175) (**Table 2**). Following the adjustment for potential confounders, a moderate and statistically significant association emerged between the disease status of subjects and the composition of microbial loads, as well as the copy number of miRNAs (R2 = 0.035, *p* = 0.046) (**Figure 1**).

**Table 2:** Absolute value of periodontal pathogens in saliva and oncomiRNAs in blood samples of PC patients and controls assessed by real-time PCR.

Variable	Pancreatic cancer group	Non-cancer group	p-value
Periodontal pathogens (Log_10_^CFU/µl^); presence/absence (n)			
*P. gingivalis* ^*^	2.02 [SD: ± 1.88]; 25/16	1.06 [SD: ± 2.08]; 9/31	0.034
*A. actinomycetemcomitans* ^*^	1.27 [SD: ± 0.91]; 35/6	1.58 [SD: ± 0.85]; 33/7	0.128
OncomiRNAs (_Log10_^copy/µl^)			
miR-21^*^	7.71 [SD: ± 2.50]	6.90 [SD: ± 2.79]	0.175
miR-155^*^	5.16 [SD: ± 3.25]	3.75 [SD: ± 2.59]	0.034

^*^Analysis was based on *t*-test and results were presented as mean ± SD.

### Biomarker associations, stratified risk analyses, and predictive model performance

Elevated levels of *P. gingivalis* were associated with a higher risk of PC (adjusted OR = 1.43 and 95% CI 1.04 to 1.96). Overexpression of miR-21 was also associated with an increased risk of PC (adjusted OR = 1.33 and 95% CI 1.04 to 1.70). In contrast, loads of *A. actinomycetemcomitans* and miR-155 were not significantly associated with the risk of PC (**Supplementary Table S2**). In analyses stratified by age, adjusted OR for *P. gingivalis* and miR-155 in relation to PC risk were 1.67 (95% CI 1.17 to 2.39) and 1.44 (95% CI 1.13 to 1.84) among individuals younger than years old, respectively. Among females, enrichment of *P. gingivalis* was associated with a higher risk of PC (adjusted OR = 2.31 and 95% CI 0.98 to 5.47) compared to males (adjusted OR = 1.36 and 95% CI 0.98 to 1.90). In analyses stratified by smoking status, no significant differences in the risk of PC were observed among ever smokers for the selected biomarkers. Notably, among individuals with type 2 diabetes, a greater abundance of *A. actinomycetemcomitans* was associated with an approximately 3-fold increased risk of PC (adjusted OR = 3.66, 95% CI 0.47 to 6.68), compared to the risks associated with elevated levels of *P. gingivalis* (adjusted OR = 1.16, 95% CI 0.60 to 2.24) (**Table 3**). However, within non-diabetic individuals, only *P. gingivalis* was related to the rise of PC risk (adjusted OR = 1.67, 95% CI 1.05 to 2.66).

**Table 3:** Selected biomarkers^┼^ and risk of pancreatic cancer, stratified by age, sex, smoking, and diabetes between pancreatic cancer cases and non-cancer controls.

Biomarkers (Log_10_^Absolute quantification value)^	Pancreatic cancer versus non-cancer		
	OR^*^	95% CI^*^	*p-value* ^*^
P. gingivalis			
Age<50	1.67	1.17-2.39	0.005
Males	1.36	0.98-1.90	0.064
Females	2.31	0.98-5.47	0.055
Never smoker	1.48	1.02-2.14	0.036
Ever smoker	1.45	0.83-2.52	0.185
Non-diabetic	1.67	1.05-2.66	0.029
Diabetic	1.16	0.60-2.24	0.654
A. actinomycetemcomitans			
Age<50	0.35	0.15-0.85	0.02
Males	0.43	0.15-1.20	0.109
Females	0.48	0.16-1.48	0.206
Never smoker	0.53	0.21-1.32	0.176
Ever smoker	0.55	0.18-1.65	0.289
Non-diabetic	0.30	0.08-1.12	0.076
Diabetic	3.66	0.47-6.86	0.211
miR-155			
Age<50	1.44	1.13-1.84	0.003
Males	1.23	0.95-1.59	0.106
Females	1.78	1.10-2.88	0.019
Never smoker	1.38	1.08-1.75	0.008
Ever smoker	1.12	0.72-1.72	0.608
Non-diabetic	1.14	0.79-1.64	0.465
Diabetic	1.45	0.95-2.21	0.082

┼ Logarithmic absolute values of *P. gingivalis*, *A. actinomycetemcomitans*, and miR-155 in the models were mutually adjusted.

*Values of ORs, 95% CIs, and p-values from LASSO logistic regression models after controlling the random effect of potential covariates (age, sex, and BMI).

BMI, body mass index.

**Figure 2:**
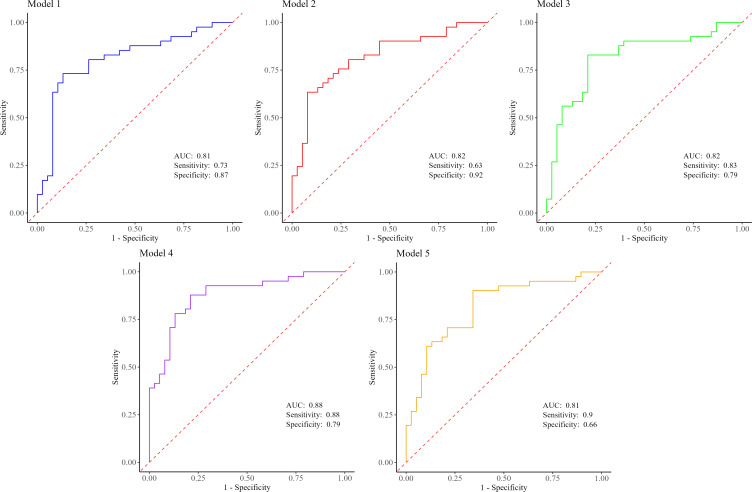
Predictive performance estimates between PC patients and non-cancer controls for model 1 (Pg+Aa+miR-155+BMI), model 2 (Pg+Aa+miR-21+miR-155+BMI), model 3 (Pg+Aa+miR-21+miR-155), model 4 (Pg+Aa+miR-21+miR-155+diabetes), and model 5 (Pg+Aa+miR-21+miR-155+smoking). PC, pancreatic cancer; Pg, *Porphyromonas gingivalis*; Aa, *Aggregatibacter actinomycetemcomitans*; BMI, body mass index.

To evaluate the performance of the biomarkers of interest, five models were constructed (**Figure 2**). The first model, based on LASSO feature selection, achieved an AUC of 0.080 (95% CI 0.707 to 0.909), with a sensitivity of 73.1% and a specificity of 86.8%. Intriguingly, model 4, which incorporated our four main biomarkers along with diabetes status, demonstrated the highest AUC of 0.878 (95% CI 0.802 to 0.955), indicating that these factors together predict the occurrence of PC with the highest accuracy (0.835). Meanwhile, model 5 exhibited the highest sensitivity (90.2%), and model 2 showed the highest specificity (92.1%) (**Figure 2**, **Supplementary Table S3**).

## Discussion

*P. gingivalis* and *A. actinomycetemcomitans* play pivotal roles as keystone pathogens within the oral microbiome, contributing significantly to the onset of periodontal disease and dental deterioration (Costalonga and Herzberg, 2014). Prior research has consistently highlighted that a history of periodontal disease and tooth loss correlates with a heightened likelihood of developing PC (Hujoel et al., 2003; Stolzenberg-Solomon et al., 2003; Michaud et al., 2007; Hiraki et al., 2008; Ahn et al., 2012). The present case-control investigation revealed a significant association between the salivary load of *P. gingivalis* and the expression level of miR-155 in individuals suffering from PC compared to controls. According to our results, among the two examined pathogens, *P. gingivalis* was significantly associated with a notable OR of 1.43 after adjusting for potential confounders, including age, gender, diabetes status, and smoking. Interestingly, higher levels of *P. gingivalis* in females were related to the highest increase in the risk of PC by nearly two-fold. Subsequently, individuals younger than 50 years old and non-diabetic individuals were at a heightened risk compared to other subgroups following an enrichment of *P. gingivalis*. Among studies conducted on the oral microbiome, three nested case-control studies have investigated the relationship between PC and *P. gingivalis* (Michaud et al., 2007; Fan et al., 2018; Petrick et al., 2022). Two of these studies focused on samples obtained from the mouthwash of patients before the onset of PC (Fan et al., 2018; Petrick et al., 2022). According to previous findings, the risk of developing PC in the presence of *P. gingivalis* increased by 1.60 (Fan et al., 2018). Additionally, never smokers and never drinkers were reported to have the highest risk of developing the disease for carriage of *P. gingivalis* (Fan et al., 2018; Petrick et al., 2022). In another prospective study conducted on blood samples from 405 individuals prior to developing PC, it was revealed that in samples where the level of antibodies against *P. gingivalis* exceeded 200 ng/ml, the risk of PC doubled (Michaud et al., 2007). Our findings are consistent with prior research indicating an association between the presence of *P. gingivalis* and the incidence of PC. However, in previous studies, the odds ratio was either based on the presence versus absence or relative abundance of this pathogen, whereas in our studies, the observed association was based on the number of *P. gingivalis* cells present in salivary samples.

Another keystone pathogen in the oral cavity is *A. actinomycetemcomitans*, which has yielded conflicting results. While one study revealed an association with an OR of 2.20 (Fan et al., 2018), both our investigation and other research failed to show a significant correlation with the risk of developing PC (Michaud et al., 2007; Petrick et al., 2022). Previous investigations have explored the intriguing aspect of assessing the presence of this periodontal pathogen in relation to PC risk within different subgroups. Noteworthy odds ratios were only significant within the subgroup of individuals who reported consuming alcohol, where the presence of bacteria was linked to an almost threefold increase in PC risk (Fan et al., 2018). Notably, in our study, the majority of the population did not report a history of alcohol consumption according to self-report questionnaires. In our subgroup analysis, individuals with diabetes were discovered to have an increased susceptibility to PC associated with the sheer number of *A. actinomycetemcomitans* cells in the oral cavity (OR = 3.66). Remarkably, the wide confidence interval observed for the association between elevated *A. actinomycetemcomitans* loads and PC risk in diabetic individuals (OR = 3.66; 95% CI 0.47–6.68) warrants careful interpretation. This breadth is primarily attributable to the limited sample size within the diabetic subgroup, where only a small number of controls (n=4) were available for comparison against PC cases (n=25). Such constraints can lead to reduced statistical precision and increased susceptibility to variability in bacterial quantification data. Furthermore, the relatively low prevalence and heterogeneous distribution of *A. actinomycetemcomitans* in salivary samples may exacerbate this issue, as outliers or measurement variability in qPCR assays can disproportionately impact estimates in smaller cohorts. While this finding suggests a potential heightened risk in diabetics, larger-scale studies are essential to refine these estimates and confirm the association. The comparatively low prevalence of *A. actinomycetemcomitans* in oral samples posed a notable challenge, as highlighted in previous studies. However, in our study, we addressed this issue effectively by employing absolute quantification techniques.

In the past few decades, there has been a strenuous effort in scientific research focusing on the oral microbiome to pinpoint high-sensitivity and high-specificity biomarkers for the early and non-invasive detection of PC (Farrell et al., 2012; Lu et al., 2019; Kartal et al., 2022; Nagata et al., 2022; Chen et al., 2023). Concurrently, research endeavors have delved into exploring circulating miRNAs for similar diagnostic objectives (Wang et al., 2009; Qu et al., 2017). To the best of our knowledge, for the first time, we combined the absolute abundance of two periodontal pathogens with the copy number of two oncomiRNAs to develop predictive models for the detection of PC. Diagnosis of PC at an early stage remains a significant challenge in mitigating the impact of this malignancy. Currently, the only Food and Drug Administration (FDA)-approved biomarker for PC is serum carbohydrate antigen (CA)19-9, primarily utilized for monitoring disease progression rather than screening, due to its insufficient sensitivity and specificity (Goonetilleke and Siriwardena, 2007). Therefore, developing non-invasive markers for either screening or early detection of the disease has been catapulting interest recently. In our study, we built a predictive model that accurately predicts PC solely based on a characteristic panel of four main biomarkers. Moreover, the diagnostic power of *P. gingivalis* and *A. actinomycetemcomitans* has not been reported in any previous study. Nevertheless, to date, numerous studies have been conducted on the diagnostic power of oral microbiome profiles in PC (Hesami et al., 2025). In a study by Farrell and colleagues, which included 28 individuals with PC and 28 healthy controls (HCs), an AUC of 0.90 with a 96.4% sensitivity and 82.1% specificity was reported for *Neisseria elongata* and *Streptococcus mitis* (Farrell et al., 2012). In another model developed using data from 20 individuals with PC and 16 HCs, the ten genera identified together yielded an AUC of 0.916 (Chen et al., 2023). The third study on 30 PC patients and 25 HCs, using a combination of four genera (*Porphyromonas*, *Fusobacterium*, *Haemophilus*, and *Leptotrichia*), achieved an AUC of 0.802 with a 77.1% sensitivity and 78.6% specificity (Lu et al., 2019). In a study by Nagata and colleagues on 47 PC patients and 235 controls, two sets of oral microbiome combinations (mostly composed of *Streptococcus* species) were identified with AUC of 0.80 and 0.82 (Nagata et al., 2022). Many studies have also investigated the diagnostic power of circulating miR-21 and miR-155 for the detection of PC, yet each miRNA individually yielded an AUC not surpassing 0.78, showcasing low sensitivity but notable specificity (Wang et al., 2009; Qu et al., 2017). Likewise, upon integrating miR-21 into our LASSO-based model (model 1), the specificity escalated to 92.1%, albeit at the cost of reduced sensitivity to 63.4%. Intriguingly, within our models, the combination of four primary biomarkers along with diabetes status demonstrated the most elevated AUC of 0.878, exhibiting a specificity of 78.9% and a sensitivity of 87.8%. This performance compares favorably with the current clinical standard, the serum biomarker CA19-9, which, in the detection of PC, demonstrates moderate sensitivity and specificity (80% and 75%, respectively) (Xing et al., 2018). To provide a more direct comparison, our model's AUC of 0.878 (95% CI 0.802–0.955) is numerically higher than the pooled AUC for CA19–9 alone, reported as 0.84 (95% CI 0.80–0.87) in a meta-analysis (Xing et al., 2018). Additionally, while CA19–9 achieved a pooled sensitivity of 80% (95% CI 0.72–0.86) and specificity of 75% (95% CI 0.68–0.80), our panel offers a sensitivity of 87.8% and specificity of 78.9%. This profile suggests a potential for improved diagnostic performance, particularly through enhancing the identification of true positives and reducing missed diagnoses. Moreover, the predictive capacity of our model is further distinguished from that of models combining CA19–9 with basic clinical features, which have reported a substantially lower AUC of only 0.564 (95% CI 0.480–0.649) (Han et al., 2023). The robust discriminatory power of our non-invasive, multi-modal panel underscores its potential clinical utility for the detection of PC, as it relies on easily accessible salivary and blood samples, presenting a pathway for more accessible testing and facilitating earlier interventions that could enhance survival rates.

Despite implementing an innovative methodology for biomarker discovery, our study was constrained by several limitations. The primary limitation was the cross-sectional nature of the design, which, coupled with a one-time sample collection, prevented the determination of causality and lacked longitudinal validation for the identified biomarkers. Another limitation of this study was the small sample size, which precluded the validation and robustness evaluation of our predictive models. This is a common challenge in pilot studies of rare diseases such as PC, given its low population prevalence. Although our cohort was recruited from a single geographic region in Iran, it is important to note that Taleghani Hospital serves as a major national referral center for GI diseases, thereby capturing a more diverse patient population than a typical single-center study. However, the absence of international, multicenter data may limit the generalizability of our findings to broader, more heterogeneous populations. The final limitation was that we did not have any data on the history or clinical staging of periodontal diseases. As the salivary load of key pathogens like *P. gingivalis* is directly influenced by periodontitis severity (Shet et al., 2013; Salminen et al., 2015), this prevented us from adjusting for it as a potential confounding factor and from discerning if microbial profiles were specifically linked to PC or underlying periodontal inflammation.

## Conclusions

In summary, for the first time, this study represented the exploration of the collective diagnostic effectiveness of *P. gingivalis*, *A. actinomycetemcomitans*, miR-21, and miR-155, demonstrating heightened disease specificity and accuracy. The results implied that the proposed panel of biomarkers shows the potential to enhance non-invasive PC detection, supplementing existing markers and paving the way for cost-effective PC screening and monitoring. As a preliminary investigation, these findings require further validation. The critical next steps must include confirmation in larger, multi-center cohorts to verify the robustness and generalizability of this biomarker panel. Furthermore, to move from association to causation, future longitudinal studies are essential to establish the temporal relationship between these biomarkers and the development of PC. Building on such validation, a key translational objective will be to investigate whether combining this novel panel with CA19–9 could yield a synergistic effect, further improving the accuracy of PC prediction. Finally, we believe that these identified biomarkers might extend beyond diagnostic purposes, offering potential for varied applications in prevention and therapeutic strategies.

## Data Availability

The datasets presented in this study can be found in online repositories. The names of the repository/repositories and accession number(s) can be found in the article/**Supplementary Material**.
